# Laser-supported partial laparoscopic nephrectomy for renal cell carcinoma without ischaemia time

**DOI:** 10.1186/1471-2490-13-31

**Published:** 2013-06-20

**Authors:** Hagen Loertzer, Arne Strauß, Rolf Herrmann Ringert, Philine Schneider

**Affiliations:** 1 University Medical Center Göttingen, Department of Urology, Georg-August-University, Robert-Koch-Str. 40, 37075, Göttingen, Alemanya

**Keywords:** Laser partial nephrectomy, Laparoscopic partial nephrectomy, Renal resection without ischaemia, Ischaemia, Laser

## Abstract

**Background:**

To date, elective nephron-sparing surgery is an established method for the exstirpation of renal tumors. While open partial nephrectomy remains the reference standard of the management of renal masses, laparoscopic partial nephrectomy (LPN) continues to evolve. Conventional techniques include clamping the renal vessels risking ischaemic damage of the clamped organ. Thus, new techniques are needed that combine a sufficient tissue incision for exstirpation of the tumor with an efficient coagulation to assure haemostasis and abandon renal vessel clamping in LPN. Laser-excision of renal tumors during laparoscopic surgery seems to be a logical solution.

**Methods:**

We performed nephron-sparing surgery without clamping of the renal vessels in 11 patients with a renal tumor in exophytic position (mean size 32 mm, ranging 8–45 mm) by laser-supported LPN.

**Results:**

Regular ultrasound monitoring and insertion of a temporary drainage showed no evidence of postoperative hemorrhage. All tumors were removed with a histopathologically confirmed surrounding margin of normal renal tissue (R0 resection). Serum creatinine, hemoglobin, and hematocrit were nearly unaltered before and after surgery.

**Conclusions:**

The experience won in these patients have confirmed that laser-assisted LPN without clamping of the renal vessels could be a safe and gentle alternative to classic partial nephrectomy in patients with exophytic position of renal tumors.

## Background

To date, therapy of renal cell carcinoma (RCC) by partial nephrectomy for small and peripheral located renal tumors is a prevailing method. Due to the equal tumorspecific survival and a lower incidence of postoperative kidney malfunction (e.g. chronic renal failure, proteinuria) it has proven to be superior to radical nephrectomy [1-3]. Another important approach in kidney tumor treatment is laparoscopic partial nephrectomy. It is a safe, well described technique [4] and a particularly gentle operation method [5-8].

To achieve bloodless operating conditions, the renal vessels are usually clamped during open and laparoscopic tumor resection. Old or predamaged organs often fail to compensate the renal ischaemia caused by clamping the renal vessels [9]. Accordingly greater morbidity regarding acute and chronic renal failure compared to radical tumor nephrectomy was noted [10]. While organ preservation is achieved, diminished renal function may result from tissue hypoxia.

Warm ischaemia time seems to be extended in laparoscopic partial nephrectomy compared to open surgery [11]. As renal damage is proportional to warm ischaemia time [8,12] diminishing ischaemia might improve results of laparoscopic partial nephrectomy. Therefore, a technique is required that assures tumor excision under bloodless conditions without clamping the renal vessels. A combination of sufficient tissue incision with an efficient coagulation for assured haemostasis is essential. To date, the commonly used techniques in laparoscopic kidney surgery for cutting only partially fulfil these requirements. Klingler et al. have reviewed several new techniques and approaches that shall reduce warm ischaemia time in LPN [13]. They consider the reliability and the haemostatic performance of existing sealants and techniques used for LPN not high enough to rely on. They demand for a new technique that makes all laparoscopic intervention safe and feasible [13]. The establishment of laser in urology provides a new surgical technique that combines both. We herein present our results using laser in laparoscopic partial nephrectomy without clamping the renal vessels.

## Method

**Figure 1:**
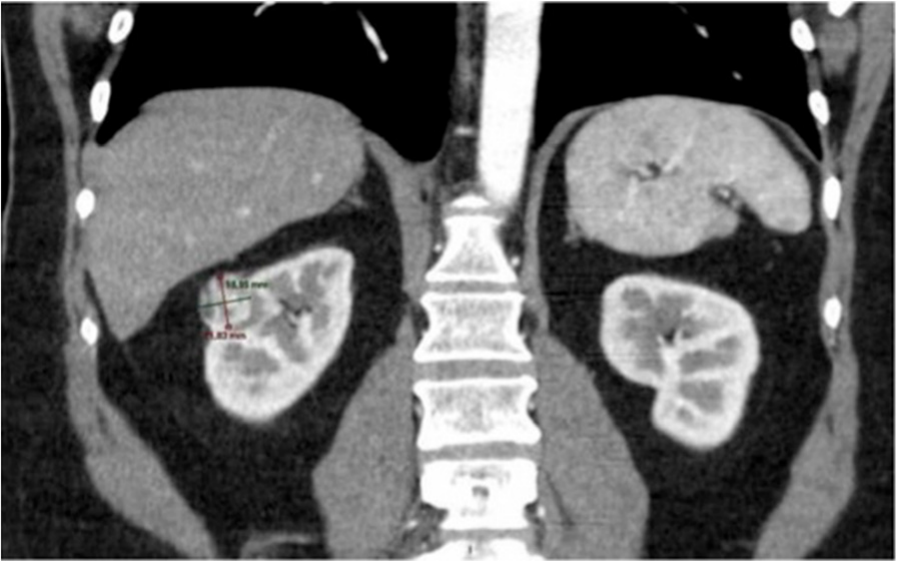
CT-scan with a 19 × 22 mm exophytic tumor of the right kidney located in the renal polar lines, 4–7 mm away from the sinus or collecting system; R.E.N.A.L.-Nephrometry Score: 7a [R(1)+E(1)+N(2)+A(a)+L(3)= 7a].

The 2.0-μm continuous wave laser (RevoLix TM) by LISA laser was used, a diode pumped solid-state laser emitting a wavelength of 2013 nm. The laser penetrates tissue to a depth of about 0.5 mm. The coagulative and ablative tissue effects are gentle. Tumor resection was performed with a safety margin of at least 2 mm. 11 patients (6 female and 5 male; age range 35 – 72, median 61 years) were treated. Included in this prospective study were patients that presented with a suspected malignant renal mass of unknown histology that had been incidentally found in routine ultrasound examination. Subsequent computer tomography scans (Figure 1) revealed that all tumors were located in the renal periphery. We applied the R.E.N.A.L. Nephrometry Score for risk evaluation and quantification of the renal masses [14]. Patients with a single kidney, centrally located renal tumors and recurrence of a former tumor were excluded. Exclusion criterias were revision procedure, ASA-score>= 3, centrally located tumor, R.E.N.A.L.-Score >9 and (functional) single kidney. All patients gave an informed consent after being given detailed information about the planned procedure. The application of the laser was approved for LISA laser under the approval no. 17-447 (UMDNS).

**Figure 2:**
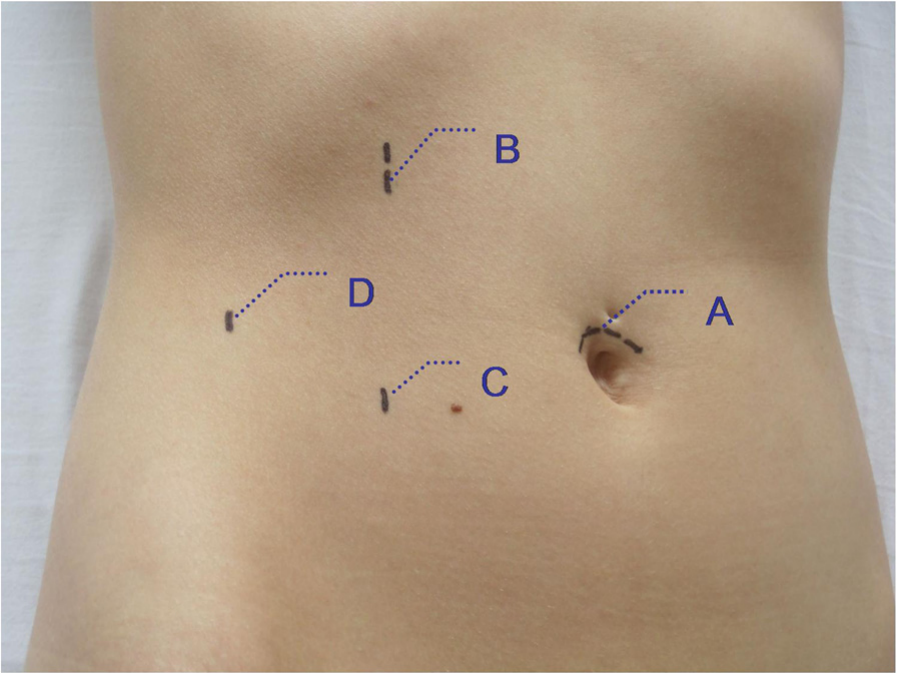
**Position of the trocars for the laparoscopic procedure. A**) Trocar for camera introduction: 12 mm Visiport™ trocar **B**) 10 mm VersaStep™ Plus trocar **C**) 5 mm VersaStep^TM^ Plus trocar, **D**) optional 5 mm VersaStep™ Plus trocar; (all trocars used are from Covidien Germany, Neustadt an der Donau).

After informed consent all patients received transperitoneal laparoscopic partial nephrectomy and laser-resection of the tumor using a power of 40 W. A transperitoneal laparoscopic technique was used, utilisizing one Visiport™ trocar (Covidien Germany, Neustadt an der Donau) and 2 – 3 VersaStep™ Plus trocars (Covidien Germany, Neustadt an der Donau), ports were located as illustrated in Figure 2. The transperitoneal procedure included complete mobilisation and preparation of the kidney with the renal hilum. No clamping of the renal vessels was performed. A directed haemostasis could be done with 15 W.

## Results

The operation time for the partial nephrectomy was less than 20 min with a mean overall operation time of 115 min (range: 85–175 min). Time needed for resection of the tumor with the laser fibre averaged 195 sec (range 100 – 320 sec). All tumors were extirpated with a safety margin of at least 2 mm surrounding the tumor tissue. No resection bleeding occurred that had to be treated by a Lahodny suture. Two patients were treated with hemostatic gauze (Tachotamp®, Ethicon, Nordheim, Germany) which was placed onto the resection area.

Mean loss of blood was 75 ml (10 – 400 ml). Postoperative follow-up was uncomplicated in all cases. Regularly performed ultrasound monitoring and drainages inserted during operation showed no secondary haemorrhage. Drainages were removed one day post operatively. Monitored serum creatinine, hemoglobin, and hematocrit were nearly unaltered pre and post-surgery. Patient’s pre- and postoperative serum creatinine differed at an average of 18 μmol/l (range: -11 to 120 μmol/l), respectively. Pre- and postoperative hemoglobin differed at a mean of 0.9 mmol/L (range: 0,3-2 mmol/L). Mean time of hospitalisation was 5 days.

All of the tumors scored below 9 points according to the R.E.N.A.L. Nephrometry score. According to the score maximum (R)adius was 2 pts (45 mm in diameter), (E)xophytic/endophytic status scored max 2 pts (more than 50% endophytic) and the distance to the collecting system was always more than 5 mm, resulting in a (N)earness score of 2 pts. A large number of the tumors treated was located crossing the polar line or in between the polar lines, (L)ocation score max. 3 pts.

Resected tumor size averaged 32 mm (range: 8 to 45 mm) (Table 1).

**Table 1:** Laser assisted LPN (WWI – with warm ischaemia time; WOI –Without ischaemia time)

**Study**	**Number of treated Patients**	**Tumor size (Ø)**	**Margin**	**Loss of blood**	**Ischaemia time (LPN)**	**Laser**	**Power**
Mattioli et al. [20]	1/9*	35 mm*	negativ	260 ml*	WWI (<30 min)	RevoLix, 2 μm diode laser; 2013 nm wavelength; continuous wave mode	15 Watt
Lotan et al. [23]	3	25 mm **	negativ	500 ml**	WOI	Holmium-YAG- Laser	0,2 J/Puls with 60 Puls/min 0,4J/Puls with 55 Puls/min 0,8J7Puls with 40 Puls/min
Khoder et al. [18]	8/13***	33 mm***	8/8 negativ	20-600***	3 WWI (1 partial; 19 & 24 min) 5 WOI	diode laser, 1318 nm wavelength; continuous wave mode	45 – 70 Watt
**Our Results**	**11**	**32 mm (8 – 45 mm)**	**negativ**	**75 (10-400 ml)**	**WOI**	**RevoLix 2 μm diode laser; 2013 nm** wavelength; continuous wave mode	**30 Watt (cutting) 15 Watt (coagulation)**

* study includes 9 patients – one laparoscopic and 8 open partial nephrectomies.

** only referring to the one case in which a renal tumor was found, the other LPN were performed on a renal cyst and a function-less pol of a kidney.

*** 13 patients were treated with laser assisted techniques– 5 open partial nephrectomies and 8 LPN, laparoscopic procedures containing 3 retroperitoneoscopic and 5 transperitoneal laparoscopic partial nephrectomies, in one open partial nephrectomy the tumor margin was found positiv.

**Figure 3:**
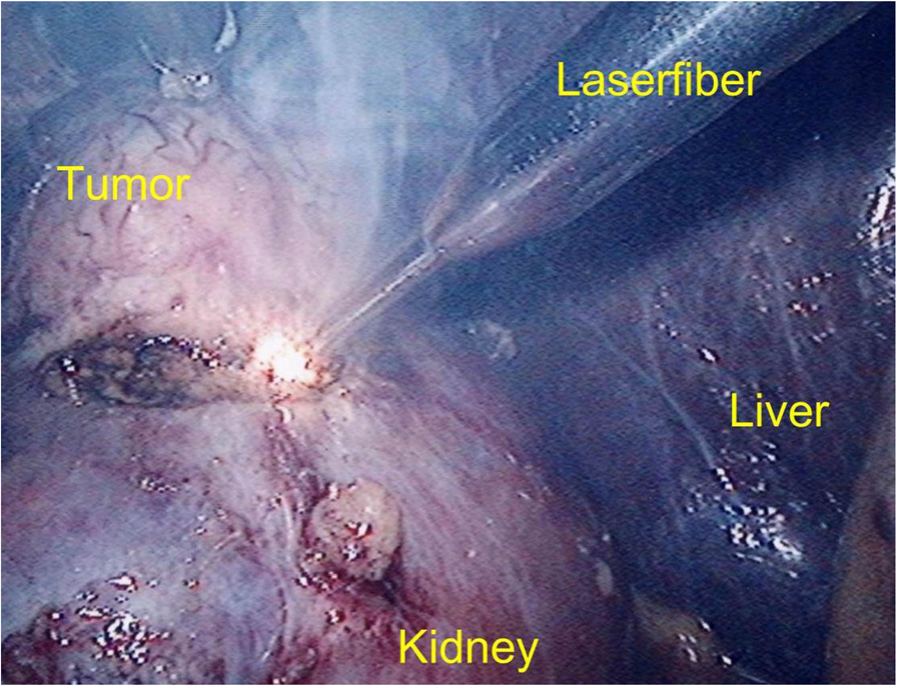
Smoke occurring due to the coagulation heat and combustion of tissue during the laser resection of a tumor of the right kidney.

**Figure 4:**
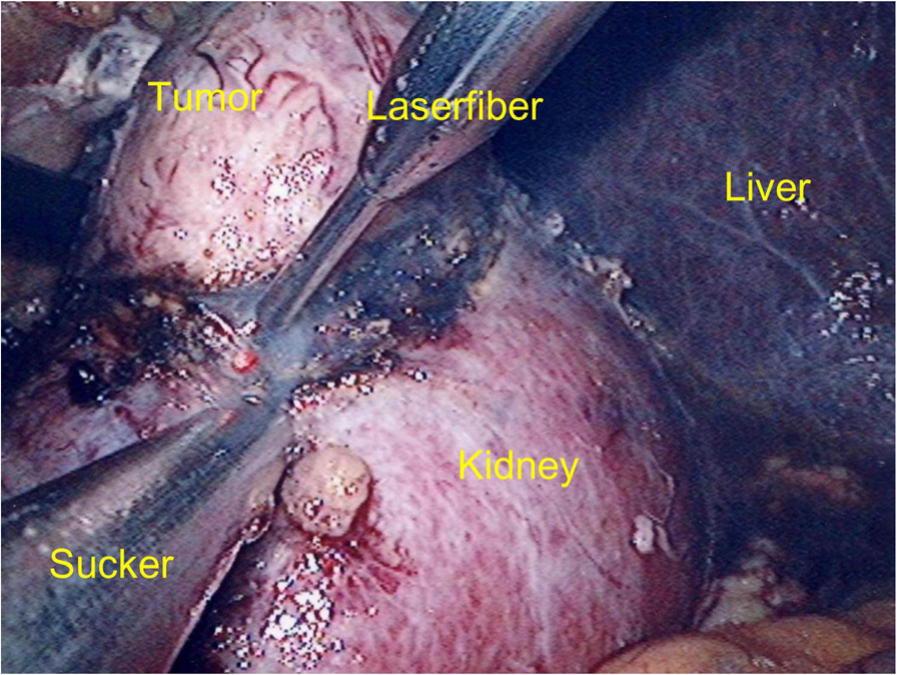
**Resection of a 3 cm diameter tumor of the right kidney with an accurate resection margin and optimal coagulation.** The smoke is eliminated with a sucker.

Histological analysis demonstrated that all malignant tumor masses (7/11 clear cell RCC) were resected with a safety margin of normal renal tissue (R0 resection) (Table 1). In two patients an angiomyolipoma was found, in one patient an oncocytom and one patient was diagnosed a haemorrhagic renal cyst. The renal pelvis remained closed throughout every operation. Due to the minimal distance between laser fiber tip and renal tissue a smooth incision and a definite discrimination between tumor and kidney as well as identification of small vessels was possible (Figures 3 and 4).

## Discussion

Conventional nephron sparing surgery includes clamping the renal vessels which is followed by ischaemia in the remnant renal tissue. Especially predamaged organs often fail to compensate the caused hypoxia which leads to an increased morbidity regarding acute and renal failure [10]. Within the first 5–8 min oxidative radicals are formed in the hypoxic tissue leading to damage [15,16]. Therefore, ischaemia should be avoided. Studies indicate that ischaemia free tumor exstirpation result in a better clinical outcome [8,12,17]. Laparoscopic partial nephrectomy itself has been shown to be a safe and especially gentle method [5-7]. However, warm ischaemia time seems to be extended compared to open surgery [11]. The use of lasers to support laparoscopic exstirpation might diminish this disadvantage in peripheral renal tumors. Whilst laser has made its way into varius fields of medicine and urology it has still remained experimental in kidney surgery. Recently, both experimental and in vivo results of laser-supported open partial nephrectomy using different laser types have been reported [17-22]. Laser-assisted LPN in turn was published only in few clinical cases [18,20,22,23]. Mattioli et al. first reported their experience with the Revolix laser in laparoscopic partial nephrectomy in one case with clampage of the renal pedicle [20]. Lotan et al. successfully performed 3 laser-assisted laparoscopic partial nephrectomies without clampage of the renal vessels using a holmium YAG laser [23]. They favourably assessed the haemostatic and cutting capacities of laser but criticize the disadvantage of smoke combustion during resection [23]. Indeed, we observed that resecting small renal tumors by means of laser-supported laparoscopy causes a lot of smoke due to the coagulation heat and combustion of tissue (Figure 3). This strongly impairs the visibility. Using an additional trocar to draw of the smoke by a sucker for laparoscopic instruments we assured a safe resection without causing a tumor burst in our study (Figure 4). Khoder et al. recently published their results of a diode laser emitting at a wavelength of 1,318 nm containing 5 cases of laser-assisted LPN and 3 laser-assisted retroperitoneoscopic partial nephrectomies with clamping the vessels in 2 cases. They studied different laser powers in laser- supported renal resection and also favourably assessed the use of the laser for exstirpation of peripheral renal tumors particularly for laparoscopic resection [18]. In all of our 11 presented cases, resection of tumor masses performed by laser-assisted LPN was possible without clamping the renal vessels which makes our series the currently largest study to this topic (Table 1).

The physical properties of lasers define and limit their mode of application in surgery: deep tissue penetration goes along with a greater risk of accidental destruction of surrounding tissue like renal or pararenal tissue or even renal hilum. Another important characteristic is the coagulation capacity. The diode-pumped solid-state laser we used in this study shows a relatively shallow penetration depth (0.5 mm). Compared to this, using a CO2- or Er: YAG-laser only allows coagulation and tissue penetrations up to a depth of 1–10 μm, enabling a better preparation of deeper stuctures. However, the coagulation capacities of these lasers are very low [22,24]. Other laser types, e.g. the Nd: YAG-laser, are not suitable for renal surgery because of their penetration depths of up to 10 cm [24]. The diode-pumped solid-state laser we used in this study possesses an optimal combination of efficient coagulation capacity and shallow tissue penetration ensuring a gentle and secure preparation of structures, e.g. tumor capsule, without cutting it accidentally [17,22]. In our study resection was performed with a power of 30 W. The used laser adjustment in a moderate cut velocity (1–3 mm/s) is sufficient for adequate manipulation (cutting as well as preparation) (Figure 4). By means of 15 W smaller vessels were successfully coagulated. Efficient and safe vascular coagulation was possible up to a vessel diameter of 1.5 mm. Using a lower power increased coagulation but less resection is attained. This agrees with the results Gruschwitz et al. published using the 2 μm continous wave laser (RevoLix™) for open laser-supported resection of small renal tumors [17].

The operative risk was relatively easily assessable by using the R.E.N.A.L. Nephrometry score. Using an online calculator (e.g. on http://www.nephrometry.com/index.htm) made this a fast and effective method [25,26]. Particularly suitable for laser-supported partial laparoscopic nephrectomy without clamping the renal vessels were tumors with a R.E.N.A.L. Nephrometry score of 4–5 pts. A higher score requires an advanced experience in laparoscopic partial nephrectomy [25].

Many critics of laser-assisted partial nephrectomy often criticize that histopathological analysis of resection margins might be not possible. Our results and those of others refute this [17]. Histological analysis in our study demonstrates that all malignant tumor masses were resected with a safety margin of at least 2 mm of healthy renal tissue. The presented clinical results are comparable to the published literature of open and laparoscopic partial nephrectomy [17,23,27].

The limitations of this experimental study are clearly set by the small cohort. For validation of this new method, multi-center studies are needed that include a long-term follow up. We herein present the results of our small experimental mono-center study.

Taking into account our results and those of others we conclude that laser-supported LPN without clamping the renal vessels seem to be a safe method for the resection of peripheral and small renal tumors with few complications. The short operating time, the minimal loss of blood and the lack of vessel clamping represent the advantage of laser-assisted LPN.

## Conclusion

Laparoscopic laser-supported partial nephrectomy without clamping the renal vessels seems to be a reasonable alternative to conventional open partial nephrectomy. This new and gentle operation method could be of particular use in the therapy of small renal tumors in elderly patients and those with an imperative indication for nephron-sparing surgery.

Our new method combines the advantages of minimal-invasive laparoscopic partial nephrectomy with the prevention of parenchymal damage by warm ischaemia. By means of absence of ischaemia and the resulting renal damage global renal function can be preserved.

## Abbreviations

RCC: Renal cell carcinoma; LPN: Laparoscopic partial nephrectomy; WWI: With warm ischaemia time; WOI: Without ischaemia time; Pts: Points.

## Competing interest

No competing financial interests exist.

## Authors’ contributions

HL conceived the study design, carried out the operative procedures, acquised, analysed and interpreted the data and made a substantial contribution to the manuscript draft. AS participated in the operative procedures and aquising the data. R-HR participated in the study design and helped to draft the manuscript. PS analysed and interpreted the data, drafted and structured the manuscript. All authors read and approved the final manuscript.

## Pre-publication history

The pre-publication history for this paper can be accessed here:

http://www.biomedcentral.com/1471-2490/13/31/prepub
